# Effects of the 2019 guideline update on lipid-lowering therapy in patients with acute coronary syndromes

**DOI:** 10.1007/s00392-025-02716-2

**Published:** 2025-07-28

**Authors:** Lukas Galli, Johannes Bernhard, Lore Schrutka, Patrick Haider, Klaus Distelmaier, Christian Hengstenberg, Konstantin A. Krychtiuk, Walter S. Speidl

**Affiliations:** 1 Division of Cardiology, Department of Internal Medicine II, Medical University of Vienna, Waehringer Guertel 18-20, 1090 Vienna, Austria; 2 Ludwig Boltzmann Institute for Cardiovascular Research, Vienna, Austria

**Keywords:** Implementation, Guidelines, Lipid therapy, Evidence-based medicine, Acute myocardial infarction

## Abstract

**Background:**

The European Society of Cardiology regularly updates its clinical practice guidelines. However, it is not well established whether guideline changes have significant effects on actual clinical practice. Therefore, we retrospectively analyzed lipid-lowering therapy at discharge after acute coronary syndrome (ACS) in a 1-year period before and a 1-year period after publication of the 2019 ESC/EAS Guidelines for the management of dyslipidaemias, respectively.

**Methods and results:**

In total, we included 691 patients who were discharged alive after AMI. A total of 354 patients were treated in the period before, and 337 after the guideline change. After the guideline change, the proportion of patients discharged on high-dose statin was higher (89.3% vs 80.5%; *p* = 0.001) and ezetimibe was prescribed more often (31.2% vs 5.9%; *p* < 0.00001) resulting in more patients being discharged on high-intensity treatment (92.9% vs. 81.6%; *p* < 0.0001). Median on-treatment LDL-cholesterol was significantly higher in the period before (65 [IQR 47 to 90] mg/dL) than after the publication of the 2019 guidelines (48 [IQR 35 to 69] mg/dL; *p* < 0.0001). The LDL-C goal of < 55 mg/dL would have been reached by 37.5% patients in the earlier period and was reached by 62.9% in the later period (*p* < 0.0001).

**Conclusions:**

The update of the 2019 ESC/EAS Guidelines for the management of dyslipidaemias was associated with a significant improvement in the prescription of high-dose statin and ezetimibe in patients after ACS. The change of the guidelines rapidly translated into clinical practice resulting in improved risk factor control in patients at very high risk.

**Graphical Abstract:**

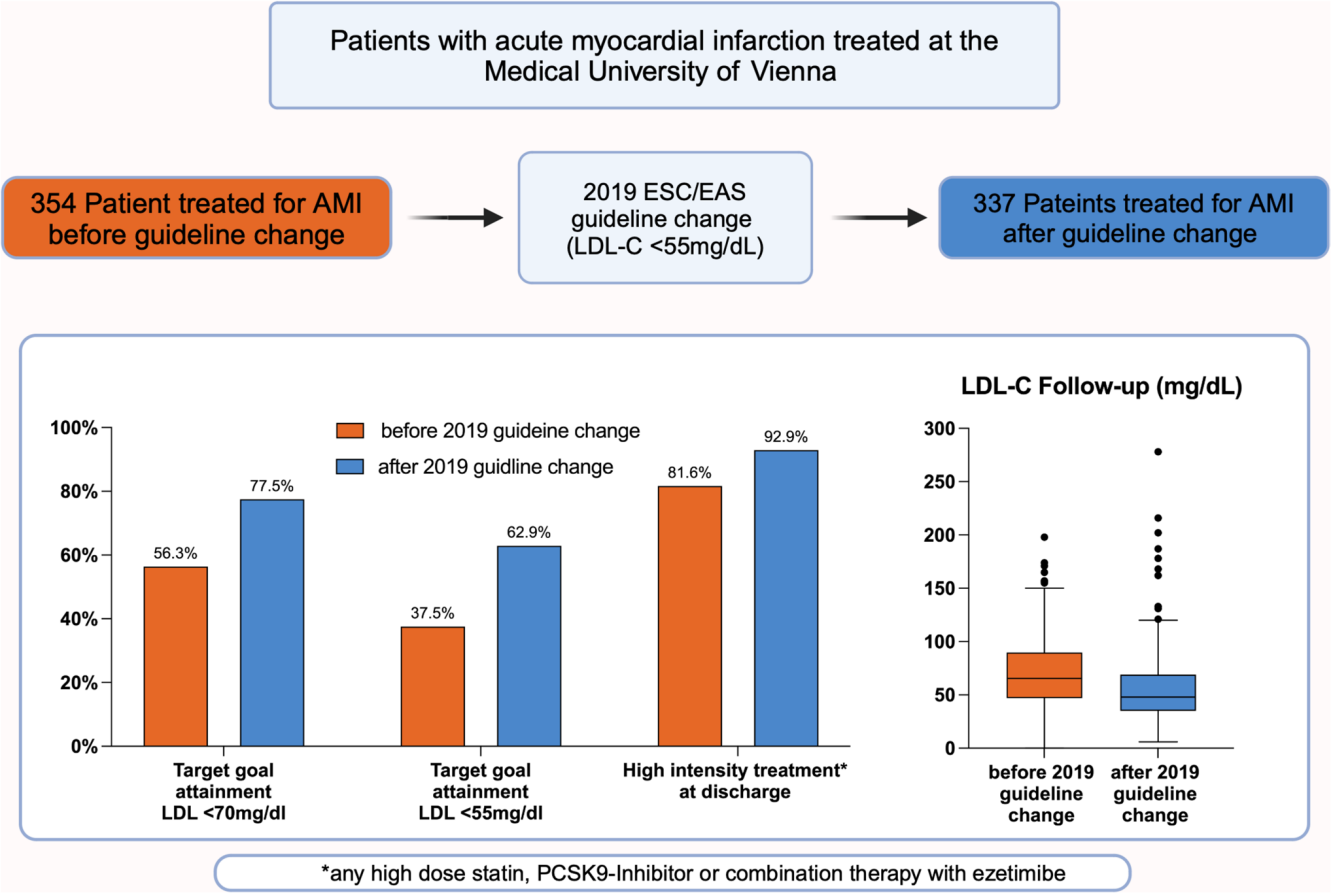

**Supplementary Information:**

The online version contains supplementary material available at 10.1007/s00392-025-02716-2.

## Introduction 

Cardiovascular disease (CVD) remains the leading cause of death in Europe [1] and globally [2]. Extensive evidence from epidemiological and genetic studies as well as intervention trials supports the causal involvement of low-density lipoprotein cholesterol (LDL-C) in the pathophysiology of atherosclerotic cardiovascular disease (ASCVD) [3]. Lipid-lowering therapy has been shown to reduce the risk of adverse cardiovascular events proportionally to the achieved reduction in LDL-C [4, 5] and thus represents a cornerstone in the treatment of ASCVD [6]. Despite the overwhelming evidence for the safety and beneficial effects of a “the lower the better” strategy for LDL-C lowering [7], especially in the setting of acute coronary syndromes [8, 9], widespread implementation in clinical practice remains unjustifiably poor, as suggested by numerous observational studies.

In the EUROASPIRE V survey including several thousand patients from 27 European countries with coronary artery disease (CAD), only 50% were treated with the recommended high-intensity statin therapy [10]. Several additional studies have supported those findings with more than 50% of ASCVD patients not receiving their recommended therapy [11, 12]. Even in leading European academic centers, most patients do not receive guideline-recommended therapy [13].

On the background of the availability of novel, safe and effective lipid-lowering therapies and accumulating evidence of a monotonic relationship between achieved LDL-C and cardiovascular events down to very low LDL-C levels, the European Society of Cardiology (ESC) and the European Atherosclerosis Society (EAS) have decided to issue novel, lower LDL-C targets in their current, *2019 ESC/EAS Guidelines for the management of dyslipidaemias*, despite the sobering results from observational studies [6]. For patients with established ASCVD, the new recommendations now suggest an LDL-C goal of < 55 mg/dL and at least a 50% reduction of baseline LDL-C.

Several barriers can prevent patients from receiving effective, inexpensive, and safe therapies which can be broadly classified as physician-, healthcare system-, and patient-related barriers [14]. In 2008, the National Heart, Lung, and Blood Institute (NHLBI) Implementation Science Work Group conducted a systematic review of evidence-based strategies for improving uptake of clinical practice guidelines on cholesterol, blood pressure, and obesity [15]. They concluded that the strategies of educational outreach visits as well as providing audit and feedback represent generally effective strategies for improving processes of care and clinical outcomes.

Herein, we aimed to assess whether more stringent LDL-C target goals recommended by the “*2019 ESC/EAS Guidelines for the management of dyslipidaemias”* improve lipid-lowering therapy and LDL-C reduction in patients with acute myocardial infarction (AMI).

## Methods

### Study population

**Fig. 1:**
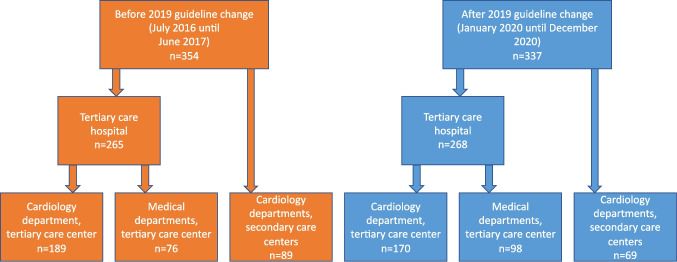
Study flowchart. All patients underwent coronary angiography at the Department of Cardiology at the Medical University of Vienna (tertiary care center) and were then transferred to the ward of the cardiology department, wards of other, non-cardiology medical departments at the Medical University of Vienna, or cardiology departments at secondary care centers within Vienna

*The 2019 ESC/EAS Guidelines for the management of dyslipidaemias* were presented and published online on September 1, 2019. We included all patients who underwent acute coronary angiography because of AMI at the Medical University of Vienna within 12 months from July 2016 until June 2017 (prior to the guideline change) and in 12 months from January until December 2020 (after the guideline change) that were discharged alive from the Medical University of Vienna or were transferred to a hospital of the “Wiener Gesundheitsverbund (Vienna Healthcare Group)” and were discharged alive there (Fig. 1).

### Blood sampling and laboratory analysis

Blood was drawn directly after admission to the medical ward or in the morning after coronary angiography when admission to the medical ward was after 2 pm. Standard laboratory measurements were analyzed in the central laboratory of the General Hospital of Vienna. Total cholesterol, high-density lipoprotein cholesterol (HDL-C), and triglycerides were measured by enzymatic methods. LDL-C was calculated using the Friedewald formula.

### Data collection

Discharge letters were reviewed and lipid-lowering medication was noted. The first lipid panel after ACS admission was collected as well as the first available on-treatment lipid panel between 1 month and 1 year after discharge, where available.

### Statistical analysis

Baseline categorical variables were summarized as counts and percentages and compared by the χ^2^ or by Fisher’s exact test as appropriate. Continuous variables were expressed as median (interquartile range). Unpaired variables were compared using the Mann–Whitney test. Paired variables were compared using the Wilcoxon rank-sum test. Two-sided *p*-values of < 0.05 indicated statistical significance. SPSS 22.0 (IBM Corporation, Armonk, NY, USA) was used for all analyses.

## Results

In total, we included 691 patients who underwent coronary angiography for AMI; 354 were included in the period between July 2016 and June 2017 (before the guideline change), and 337 patients in the 12-month period after the guideline change between January and December 2020. A total of 405 patients (58.6%) were treated for STEMI and 286 (41.4%) had a diagnosis of NSTEMI (41.4%). Of all patients, 22.7% were transferred to a secondary care hospital after coronary angiography, and 77.3% were treated and discharged from cardiology and non-cardiology medical departments at the tertiary care institution (Fig. 1). The median age was 62 years and 67.3% of patients were male. In total, 21.5% of patients had a history of previous myocardial infarction, and 23.5% had diabetes (Table 1).

**Table 1:** Baseline characteristics of the patient population according to time of inclusion

	**Before the 2019 guideline change** **(*n* = 354)**	**After the 2019 guideline change** **(*n* = 337)**	***p* value**
Age, median (IQR)	61 (53–71)	63 (54–74)	0.057
**Gender**			0.339
Male, *n* (%)	236 (65.7%)	233 (69.1%)	-
Female, *n* (%)	123 (34.3%)	104 (30.9%)	-
BMI, kg/m^2^, median (IQR)	26.6 (23.4–30.3)	26.3 (23.0–30.2)	0.665
Smoker, *n* (%)			0.533
Former, *n* (%)	34 (11.7%)	37 (12.5%)	-
Current, *n* (%)	109 (38.1%)	124 (42%)	-
Diabetes mellitus, *n* (%)	83 (23.4%)	79 (23.7%)	0.95
Family history of CAD, *n* (%)	21 (17.9%)	19 (13.1%)	0.380
Previous myocardial infarction, *n* (%)	81 (23.0%)	66 (19.9%)	0.330
PAD, *n* (%)	48 (13.6%)	40 (12.2%)	0.576
Stroke, *n* (%)	14 (4.0%)	16 (4.9%)	0.568
COPD, *n* (%)	23 (7.5%)	18 (6.2%)	0.542
CKD, *n* (%)	27 (8.8%)	34 (11.8%)	0.237
STEMI, *n* (%)	202 (57.1%)	203 (60.2%)	0.397
Total cholesterol (mg/dL), median (IQR)	182.0 (155.0–215.3)	186.0 (154.0–216.0)	0.832
HDL (mg/dL), median (IQR)	42.0 (35.0–51.0)	42.0 (36.0–54.0)	0.607
LDL (mg/dL), median (IQR) **†**	109.50 (85.8–138.0)	106.5 (76.0–134.0)	0.024
Triglycerides (mg/dL), median (IQR)	130.0 (94.5–189.0)	154.0 (114.0–238.0)	< 0.001
HbA1c (%), median (IQR)	5.7 (5.4–6.4)	5.7 (5.4–6.4)	0.769
Lipoprotein (a)	19 (7–77)	30 (8–96)	0.446
Creatinine (mg/dL), median (IQR)	1.02 (0.89–1.20)	1.00 (0.86–1.25)	0.677
Any LLT at admission	110 (31.2%)	88 (26.2%)	0.17

*IQR* interquartile range, *BMI* body mass index, *CAD* coronary artery disease, *PAD* peripheral artery disease, *COPD* chronic obstructive pulmonary disease, *CKD* chronic kidney disease, *STEMI* ST elevation myocardial infarction, *HDL* high-density lipoprotein, *LDL* low-density lipoprotein, ***†**** LDL* value calculated with Friedewald formula, *LLT* lipid-lowering therapy

### Lipid-lowering therapy (LLT) at the time of admission for the acute coronary syndrome

**Fig. 2:**
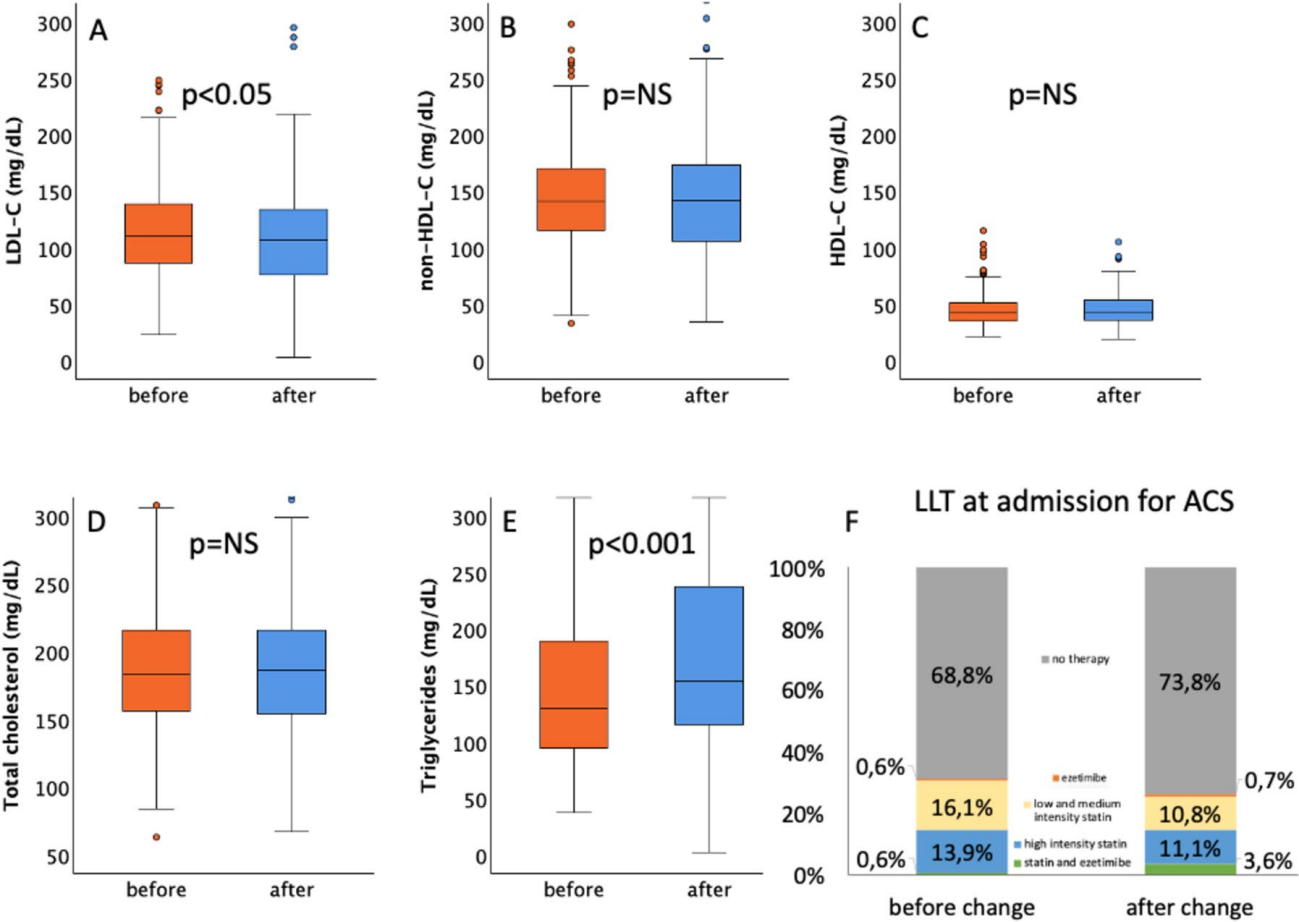
Lipid parameters and lipid-lowering therapy at admission for acute myocardial infarction before and after the 2019 guideline change. Plasma levels of LDL-cholesterol (**A**), non-HDL-cholesterol (**B**), HDL-cholesterol (**C**), total cholesterol (**D**), and triglycerides (**E**) in the period before the 2019 guideline change (orange) and after the 2019 guideline change (blue). Lipid-lowering therapy at admission (**F**). LLT Lipid-lowering therapy; ACS acute coronary syndrome

At the time of admission, a majority of the patients were without LLT (before change, 68.8%; after change, 73.8%; *p* = 0.17). There were no differences in admission-therapy regarding treatment with high-dose statins or low and medium-dose statins (Fig. 2). Although treatment with ezetimibe was very rare in both groups at the time of admission for ACS, it was significantly more common after the guideline update (4.3%) as compared to the time period before the guideline was changed (1.2%; *p* = 0.02). No patients were treated with PCSK9 inhibitors when admitted for ACS.

### Lipid parameters at the time of the acute coronary syndrome

Plasma levels of total cholesterol, non-HDL-C, and HDL-C at admission for ACS were similar in both time periods (Fig. 2). Interestingly, LDL-C was significantly lower in the period after the guideline-change (before, 110 [IQR 86 to 139] mg/dL vs. after, 106 [IQR 76 to 134] mg/dL; *p* < 0.05) and triglyceride levels were markedly higher after publication of the new guidelines (before, 129 [IQR 94 to 189] mg/dL vs. after, 154 [IQR 114 to 238] mg/dL; *p* < 0.001).

### Lipid-lowering therapy at discharge after AMI

**Fig. 3:**
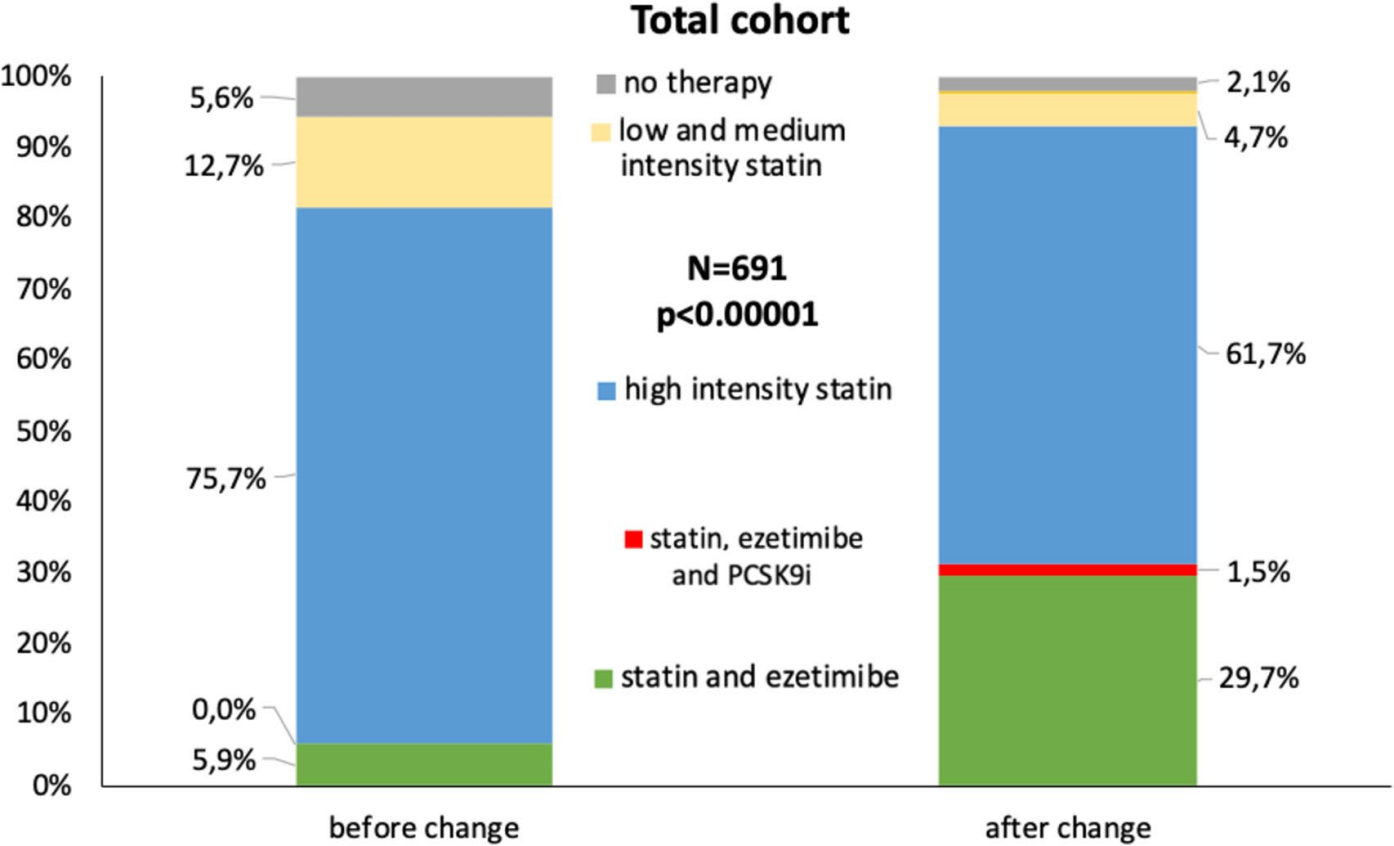
Lipid-lowering therapy at discharge before and after the 2019 guideline change in the total cohort. The proportion of combination therapy of statins with ezetimibe or PCSK9i increased significantly, whereas monotherapy with high-intensity or low and medium-intensity statins declined significantly. PCSK9i proprotein convertase subtilisin/kexin type 9 inhibitor

Although the majority of patients were discharged on LLT in both periods, significantly more patients were on treatment with LLT in the period after the guideline change (97.9%) as compared to the period before the guideline update (94.4%; *p* = 0.02; Fig. 3). The rate and intensity of statin treatment increased after the guideline update. Before the guideline change, 80.5% of patients were discharged on high-dose statins, compared to 89.3% after the guideline update (*p* < 0.001). The prescription of low and medium-dose statins decreased from 13.8 to 8.3%. Treatment with ezetimibe increased markedly from 5.9 to 31.2% (*p* < 0.00001). In the period before the new guidelines, no patients were discharged on PCSK9 inhibitors as compared to five (1.5%; *p* = 0.02) after publication of the new guidelines. Treatment with a high-dose statin, the combination therapy of any statin and ezetimibe, as well as PCSK9-inhibitor treatment, was grouped as high-intensity LLT; low and medium-dose statin or monotherapy with ezetimibe was grouped as low-intensity LLT (Supplemental Table 1). High-intensity treatment was significantly more common with the new guidelines (92.9%) as compared to the period before the update (81.6%; *p* < 0.0001).

### Comparison of new guideline implementation according to the type of center and department.

**Fig. 4:**
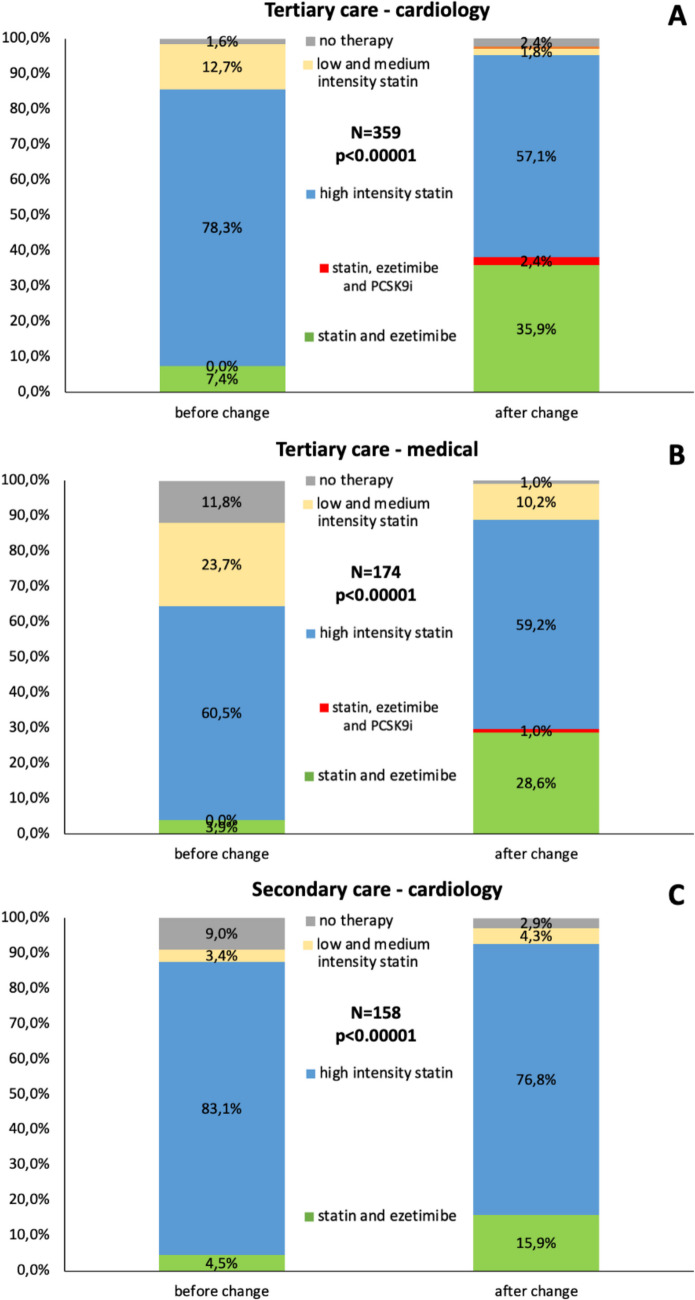
Lipid-lowering therapy at discharge according to type of center and medical department, before and after the guideline change. At hospital discharge from cardiology departments of the tertiary care center (**A**), other medical departments of the tertiary care center (**B**), and cardiology departments of the secondary care centers (**C**). PCSK9i proprotein convertase subtilisin/kexin type 9 inhibitor

Out of the 691 patients with AMI, 359 (52.0%) were treated at the cardiology and 174 (25.2) were treated at other non-cardiology medical departments of the primary PCI tertiary care center. A total of 158 patients (22.9%) were transferred to cardiology departments of secondary care centers immediately after cardiac catheterization (for flowchart, see Fig. 1). In the period before the guideline change, patients discharged from cardiology departments in both tertiary and secondary care centers had a similarly rate of high-intensity LLT (85.7% vs. 87.6%; *p* = 0.66), which was higher as compared to patients discharged from non-cardiology tertiary care medical departments (64.5%; *p* < 0.001; Fig. 4). In all three groups, the guideline change significantly affected LLT. Combination therapy with statin and ezetimibe particularly increased in tertiary care settings receiving multifaceted educational implementation efforts as compared to secondary care. After the introduction of the new guidelines, the percentage of patients discharged from tertiary care non-cardiology medical departments on high-intensity LLT at discharge significantly increased from 64.5 to 88.8% (*p* < 0.00001), resulting in a comparable rate of high-intensity LLT prescription as observed in patients treated in the cardiology departments at secondary and tertiary care (Fig. 4).

### Lipid parameters and LDL-C-goal attainment at follow-up

**Fig. 5:**
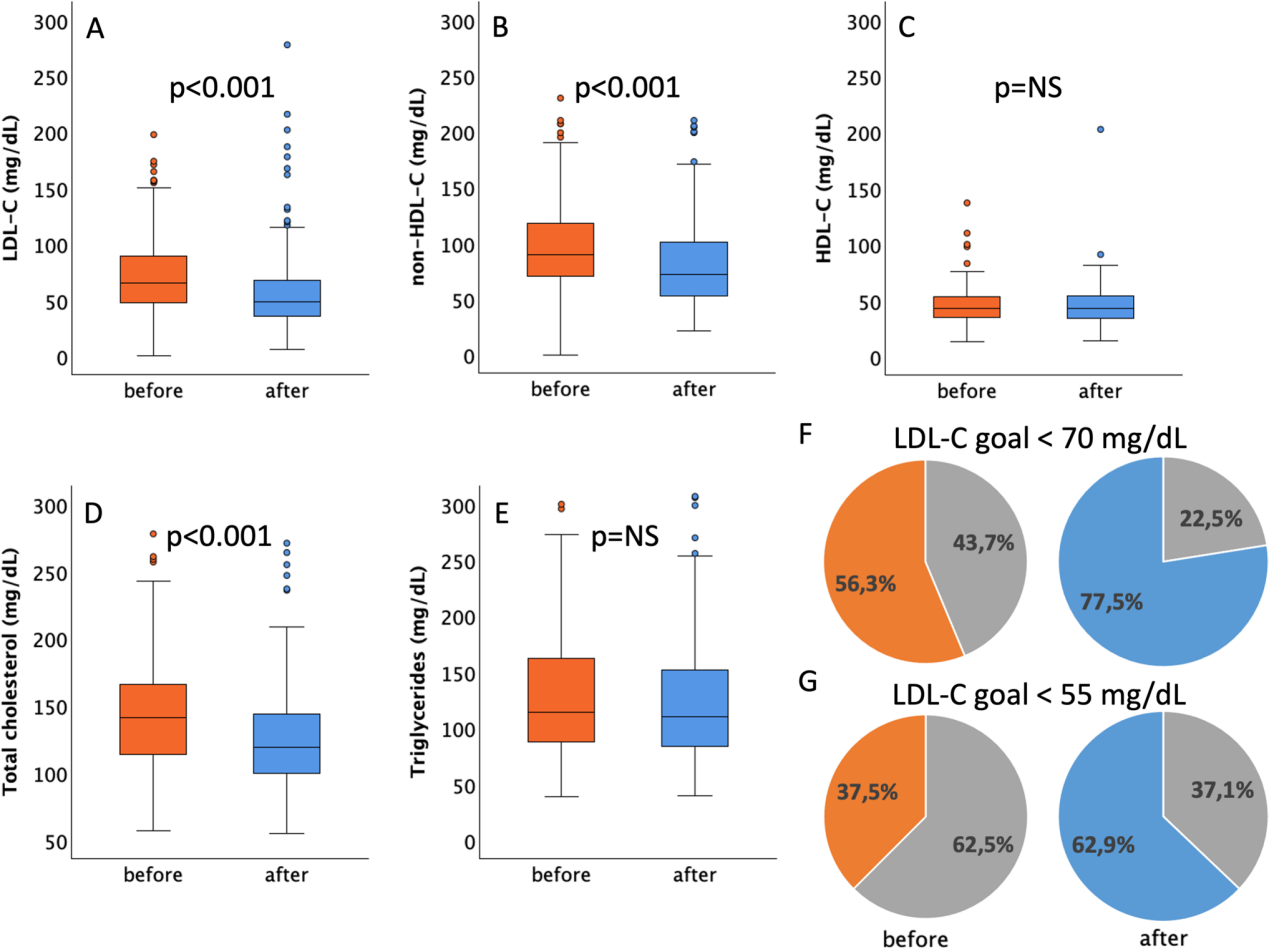
Lipid parameters and LDL-C-goal attainment at follow-up before and after the 2019 guideline change. Plasma levels of LDL-cholesterol (**A**), non-HDL-cholesterol (**B**), HDL-cholesterol (**C**), total cholesterol (**D**), and triglycerides (**E**) in the period before the 2019 guideline change (orange) and after the 2019 guideline change (blue). LDL-C-goal attainment according to the *2016 ESC/EAS Guidelines for the management of dyslipidaemias* (LDL-C < 70 mg/dL, **F**) and according to the *2019 ESC/EAS Guidelines for the management of dyslipidaemias* (LDL-C < 55 mg/dL, **G**). Lipid parameters at follow-up between 1 month and 1 year after discharge were available in 295 (42.7%) patients

Lipid parameters at follow-up between 1 month and 1 year after discharge were available in 295 (42.7%) patients. In the periods before and after the guideline updates, blood sampling was available at 155 (IQR 63–281) and 105 (IQR 59–183) days after the acute event, respectively. Patients with follow-up at our clinic and with follow-up lipid parameters available more often had diabetes, a history of MI, peripheral artery, and chronic kidney disease. Baseline levels of total cholesterol and triglycerides were higher compared to patients without available follow-up parameters (supplemental Table 2). After publication of the new guidelines, LDL-C, non-HDL-C, and total cholesterol at follow-up were significantly lower as compared to the period before the guidelines were updated (Fig. 5). Median LDL-C levels in the period before the guideline update were 65 (IQR 47 to 90) mg/dL as compared to 48 (IQR 35 to 69) mg/dL after the guidelines update (*p* < 0.0001). In the period before the guidelines were updated, 56.3% of patients reached the goal recommended by the *2016 ESC/EAS Guidelines for the management of dyslipidaemias* of LDL-C < 70 mg/dL as compared to 77.5% of patients in the later period (*p* < 0.001). The LDL-C goal of < 55 mg/dL as recommended by the *2019 ESC/EAS Guidelines for the management of dyslipidaemias* would have been reached by 37.5% patients in the earlier period and was reached by 62.9% in the later period (*p* < 0.0001).

## Discussion

We conducted a retrospective analysis to assess the impact of the 2019 ESC/EAS guideline update on guideline adherence in a single-center registry of patients undergoing coronary angiography for AMI at the Medical University of Vienna. The study compared two groups of AMI patients: One group treated before the guideline update and one afterward. Our findings suggest that patients benefited from the updated guidelines, receiving high-intensity, high-dose LLT more frequently and achieving lower LDL-C levels at follow-up.

With the introduction of ezetimibe and PCSK9 inhibitors and the growing evidence stemming from cardiovascular outcome trials on the possibility and the benefits of achieving very low levels of LDL-C with virtually no lower limit described [7, 9, 16, 17], the 2019 ESC/EAS guidelines for dyslipidemia management introduced an ambitious LDL-C target goal of < 55 mg/dL for very high-risk patients. Thereby, the guideline committee raised the bar from the previous < 70 mg/dL target, a goal that has already proven to be challenging, with multiple studies highlighting disappointingly low adherence to these targets [10, 11, 18]. This raises the question of whether the 2019 guideline changes significantly benefit clinical practice.

With the results of our analysis, we could provide evidence that not only more patients received adequate post-ACS LLT under the new guideline regimen but also that much higher rates of patients achieving target goals were manageable in a clinical real-life setting.

Comparing our findings to the Da Vinci study [11], 54% of patients reached the 2016 target goal vs. 56.3% in our cohort, and 33% achieved the 2019 guidelines vs. 37.5% in our cohort. Both groups are pretty comparable in LDL-C target goal attainment and consist of patients treated before the 2019 guideline change. This is on par with the EUROASPIRE V survey [10] suggesting that 71% of patients did not achieve their LDL-C target goals in Europe. Looking at our findings, however, after the 2019 guideline change, 77.9% of patients reached conservative LDL-C target goals and 62.5% reached the new LDL-C target. This is an impressive improvement, especially considering that the SANTORINI study [12] showed again that up to 80% of patients did not reach the 2019 high and very high-risk targets. In a smaller survey study from leading European institutions, LDL-C targets were achieved in less than half of all patients post ACS and an even lower proportion of patients achieved both an LDL-C < 55 mg/dL and a LDL-C reduction by at least 50%. The aforementioned studies still represent selected populations, as they were all either part of active registries, surveys, or obtained from tertiary care teaching institutions. Several analyses from large insurance networks in the USA suggest that approximately half of all patients with a diagnosis of ASCVD are not even treated with a statin at all [19, 20].

Such gross underuse of highly effective but inexpensive and readily available drugs represents one of the most pressing issues in modern cardiovascular medicine. Factors hindering widespread implementation of drugs for cardiovascular disease prevention include barriers on the healthcare system, clinician, and patient level [14]. A variety of implementation strategies to improve the use of effective and proven therapies have recently been tested with varying success, including nudges and multifaceted coordinated approaches [21].

The publication of the 2019 guidelines resulted in a significantly higher proportion of patients discharged on dual lipid-lowering therapy with a statin and ezetimibe. Furthermore, while high-intensity lipid therapy was significantly less well-established in non-cardiology departments than in patients treated in cardiology departments in the first period of our study, after the publication of the 2019 guideline update, the above-mentioned differences vanished. After the update of the 2019 guidelines and the implementation of the bundle to improve guideline adherence, high-intensity LLT at discharge has become aligned between the medical and cardiology departments.

The high rate of ezetimibe prescribed at discharge is a contributing factor to our positive results on guideline adherence. Based on the positive results of the IMPROVE IT trial showing superiority for an upfront combination therapy of a statin and ezetimibe [22], the predictable LDL-C response to treatment, the “the lower the better” principle, and the widespread availability as generic medications suggest that initial combination therapy seems to be a reasonable approach in patients after ACS [8] and has been mentioned in the most recent ACS guidelines [23]. It has been shown that patients benefit from a combination of statins and ezetimibe in CV outcome irrespective of baseline LDL-C [24] and LLT naïve high and very high-risk patients are more likely to achieve their LDL-C target goal if combination therapy is immediately established at baseline [25]. Recent data from a national multicenter observational registry in Poland, for instance, suggested that upfront combination therapy is superior as compared to statin monotherapy with regard to all-cause mortality [26]. Considering poor target goal attainment in post-ACS patients, adding ezetimibe to discharge therapy may be beneficial regardless of baseline LDL-C in post-AMI patients.

Interestingly, baseline triglyceride levels were significantly higher and LDL-C levels were significantly lower in the period after the guideline change. This may be explained, at least in part, by the fact that blood sampling was performed fasting before the guideline change, which was not mandatory in the new guidelines leading to a practice change. A higher number of non-fasting blood sampling may have resulted in higher triglyceride levels leading to lower LDL-C values as they were calculated by the Friedewald formula [27].

## Limitations

Several limitations warrant discussion. As our analysis was confined to data from a single center, the generalizability of our results to other institutions or broader patient populations is limited. However, a significant portion of our patient cohort received follow-up care after coronary angiography in secondary care clinics. Another key limitation is that we could only retrieve follow-up laboratory measurements in 42.7% of patients. This is primarily because most patients received ongoing care from community-based physicians outside our data network. Notably, patients with follow-up at our institution were more likely to be a high-risk population with comorbidities. Therefore, lipid parameters at follow-up cannot be generalized to the total population. Follow-up lipid parameters were obtained in a time period from 1 month to 1 year after discharge. We cannot exclude that at later time points, LLT could have been more intensified or, in contrast, lipid levels could be higher due to a lower adherence to LLT at later time points. However, we used the initial LDL-C values available after discharge, prior to any subsequent outpatient optimization.

## Conclusion

The newly set target goals of LDL < 55 mg/dL for very high-risk patients following AMI in the 2019 EAS/ESC guidelines were associated with improved LLT at discharge, LDL-C at follow-up, and target goal attainment. The change of the guidelines rapidly translated into clinical practice resulting in improved risk factor control in patients at very high risk.

## Supplementary Information

Below is the link to the electronic supplementary material.

Supplementary File 1 (DOCX 890 KB)

## Data Availability

The data underlying this article will be shared on reasonable request to the corresponding author.

## Declarations

### Use of AI and AI-assisted technologies statement

No AI or AI-assisted technologies have been used.
