# Supplementary material for: Effects of the 2019 guideline update on lipid-lowering therapy in patients with acute coronary syndromes
Source: Clin Res Cardiol. 2025 Jul 28;115(2):277–87. doi: 10.1007/s00392-025-02716-2 (PMC12823727; doi:10.1007/s00392-025-02716-2)
Supplement: Supplementary file 1 — (DOCX 890 KB) [file 392_2025_2716_MOESM1_ESM.docx]

**Supplemental figure 1**

**Supplemental Table 1** Definition of high-intensity and low-intensity lipid-lowering therapy

| **High intensity lipid lowering therapy** |
| --- |
| Atorvastatin 40 mg, 80 mg |
| Rosuvastatin 20 mg, 40 mg |
| Alirocumab 150 mg |
| Evolocumab 140 mg |
| Combination of any statin plus ezetimbe |
| Combination of any statin plus PCSK9 inhibitor |
| Combination of ezetimibe plus PCSK9 inhibitor |
| Combination of any statin, ezetimibe plus PCSK9 inhibitor |
| **Low intensity lipid lowering therapy** |
| Atorvastatin 10 mg, 20 mg |
| Fluvastatin 20 mg, 40 mg |
| Lovastatin 10 mg, 20 mg, 40 mg |
| Pravastatin 10 mg, 20mg, 40 mg |
| Rosuva 5 mg, 10 mg |
| Simvastatin 10 mg, 20 mg, 40 mg, 80 mg |
| Ezetimibe 10mg |

**Supplemental Table 2**

|  | **Follow-Up LDL-C available**  **(n = 295)** | **Follow-Up LDL-C not available**  **(n = 396)** | **p Value** |
| --- | --- | --- | --- |
| Age, median (IQR) | 62 (52-72) | 62 (54-72) | 0.545 |
| **Gender**  Male, n (%)  Female, n (%) | 191 (64.7%)  104 (35.3%) | 275 (69.4%)  121 (30.6%) | 0.192  -  - |
| BMI, kg/m^2^, median (IQR) | 26.8 (23.8-30.5) | 27.1 (24.7-31.2) | 0.075 |
| Smoker, n (%)  former, n (%)  current, n (%) | 40 (14.7%)  116 (42.5%) | 30 (9.7%)  119 (38.4%) | **0.047**  -  - |
| Diabetes mellitus, n (%) | 81 (27.5%) | 81 (20.6%) | **0.036** |
| Family history of CAD, n (%) | 14 (11.1%) | 26 (18.7%) | 0,137 |
| Previous myocardial infarction, n (%) | 78 (26.4%) | 70 (18%) | **0.019** |
| PAD, n (%) | 56 (19%) | 34 (8.8%) | **<0.001** |
| Stroke, n (%) | 17 (5.8%) | 13 (3.4%) | 0.133 |
| COPD, n (%) | 26 (9.6%) | 14 (4.3%) | **0.010** |
| CKD, n (%) | 39 (14.4%) | 21 (6.4%) | **0.001** |
| STEMI, n (%) | 170 (57.6%) | 235 (59.3%) | 0.650 |
| Total cholesterol (mg/dl), median (IQR) | 190 (157-223) | 181.5 (152.75-213) | **0.006** |
| HDL (mg/dl), median (IQR) | 43 (36-53.75) | 42 (35-52) | 0.110 |
| LDL (mg/dl), median (IQR) **†** | 109.2 (80.5-138) | 107.4 (80.8-134.2) | 0.232 |
| Triglycerides (mg/dL), median (IQR) | 150 (108-226) | 137.5 (100.8-199.3) | 0.062 |
| HbA1c (%), median (IQR) | 5.7 (5.4-6.6) | 5.7 (5.4-6.3) | 0.260 |
| Lipoprotein (a) | 30 (8-110) | 20 (8-64) | 0.066 |
| Creatinine (mg/dl), median (IQR) | 0.97 (0.81-1.16) | 0.91 (0.79-1.1) | **0.039** |

*IQR interquartile range, BMI body mass index, CAD coronary artery disease, PAD peripheral artery disease, COPD chronic obstructive pulmonary disease, CKD chronic kidney disease, STEMI ST-elevation myocardial infarction, HDL high-density lipoprotein, LDL low-density lipoprotein,* ***†*** *LDL value calculated with Friedewald formula;*
